# Anxiety and depression symptoms and alcohol use among adolescents - a cross sectional study of Norwegian secondary school students

**DOI:** 10.1186/s12889-017-4389-2

**Published:** 2017-05-23

**Authors:** Espen Lund Johannessen, Helle Wessel Andersson, Johan Håkon Bjørngaard, Kristine Pape

**Affiliations:** 1 Department of Public Health and Nursing, Norwegian University of Science and Technology, 7491 Trondheim, Norway; 2 Kongsberg Hospital, Vestre Viken HF, Kongsberg, Norway; 3 St. Olav’s University Hospital Trondheim, Forensic Department and Research Centre Bröset, Trondheim, Norway; 4 Department of Research and Development, Clinic of Substance Use and Addiction Medicine, St. Olav’s University Hospital, Trondheim, Norway

**Keywords:** Alcohol, Adolescence, Depressive symptoms, Anxiety symptoms

## Abstract

**Background:**

We investigated the associations between symptoms of anxiety and depression and alcohol drinking behavior among adolescents, by focusing on the importance of symptom load, as well as gender differences.

**Methods:**

Data was derived from a cross-sectional school based survey among adolescents in upper secondary schools in Norway. Among other variables adolescents reported on symptoms of anxiety and depression, time of onset and extent of alcohol use. The sample consisted of 6238 adolescents aged 16–18 years. We estimated prevalence of alcohol drinking behaviors in relation to severity of symptoms of anxiety and depression.

**Results:**

Higher levels of depression symptoms were associated with earlier onset of alcohol use, more frequent consumption and intoxications. The associations between anxiety and depression symptoms and early drinking onset were stronger for girls than for boys. Higher levels of anxiety symptoms were only associated with alcohol consumption among girls.

**Conclusions:**

Boys and girls with depressive symptoms and girls with anxiety symptoms are more likely to have unhealthy patterns of alcohol drinking. Preventive strategies at all levels could possibly profit from a common approach to mental health and alcohol use, in particular for girls in mid-adolescence.

## Background

Increasing levels of mental symptoms and alcohol consumption are each considered to be features of normal development during adolescence (defined by WHO as the period from ages 10 to 19) [1, 2]. However, both factors carry an increased risk of developing health problems later in life [3]. The combination of anxiety and depression symptoms and harmful alcohol consumption is commonly observed in young people [4], also among those who do not satisfy the diagnostic criteria of neither mental disorders nor alcohol abuse [5]. This combination may be particularly harmful for the adolescent brain, influence behavior and increase the future risk of mental disorders and substance abuse [6]. How and why symptoms of anxiety and depression and harmful alcohol consumption often occur simultaneously is not well understood, including possible causal mechanisms [7, 8]. The self-medication hypothesis, explaining the relationship by suggesting that alcohol is used to relieve emotional stress, is suggested to be the approach with the greatest support in the research literature [9, 10].

Interestingly, both the trends in the prevalence of mental health problems and alcohol drinking patterns among Norwegian adolescents seem to have changed over the last two decades. While there is evidence to suggest increased prevalence of depressive symptoms over the last two decades, and in particular among girls [11–13], different studies have suggested that adolescents tend to drink less alcohol [1]. Norwegian adolescents tend to have the same drinking patterns as adolescents in other Nordic countries, characterized by heavy episodic drinking and a more equal drinking pattern between boys and girls than in other European countries [1, 14]. However, a recent decrease in both heavy episodic drinking and the amount of alcohol consumed has been observed across the Nordic countries [1]. Existing literature indicates that the association with alcohol consumption may be stronger for depression symptoms than for anxiety symptoms [15, 16], and perhaps more pronounced for girls than for boys [4, 17]. However, support for this from recent studies on a general Norwegian youth population is lacking. Also, we lack knowledge on the association between the two factors within the ranges of mild-to-moderate levels of symptoms and common alcohol consumption patterns. This calls for updated knowledge on the field, and particular attention to sex differences. The aim of this study was thus to examine the relationship between anxiety and depression symptoms and alcohol consumption in a representative population of Norwegian adolescents. We also investigated the possible sex differences regarding this relationship, and whether the relationship was different for symptoms of anxiety and depression.

## Methods

### Sample and data collection

Ungdata is a quality assured and standardized system designed to conduct local surveys of adolescents in Norway [18]. The online questionnaire is administered anonymously at school, during a school hour. Participation is voluntary and based on informed consent. The Ungdata survey is an important source of information on adolescent health and well-being, including mental health and substance use, and a tool for monitoring the psychosocial environment, both at the municipal and the national level. The survey is conducted by the Norwegian Social Research (NOVA). It is financed by the Norwegian Directorate of Health, The Ministry of Children, Equality and Social Inclusion, and the Ministry of Justice and the Public Security, and may be ordered free of charge by the school administrators in the municipalities. The scheme includes about 150 questions that are similar across all surveys. Furthermore, the schools may choose an extended version of the survey, including additional sets of questions regarding mental health. Since it started in 2010, the Ungdata-survey has been conducted in many lower secondary schools all over the country. From 2013, some upper secondary schools were also included. For more information on Ungdata see [18].

In total, 6938 pupils from 12 upper secondary schools completed the additional version of the Ungdata-survey in 2013 (which included the questions on anxiety symptoms). These schools were located in different municipalities, and different geographical areas of the country, representing both urban and rural areas. The response rate at school-level varied between 50 and 82%, and the overall response rate among students in secondary schools in the Ungdata surveys is reported to be 66% [13]. Reasons for non-response was either that the adolescents were absent from school when the survey was conducted or that they did not want to participate. Participants in the third year of upper secondary school (13th year of school) were excluded from main analyses as they comprised a small sub-sample of 263 pupils from only a few of the rural schools/municipalities. We excluded respondents with missing data on gender (*n* = 281) since all our analyses were stratified on this variable. The main analyses were performed on a sample of 6238 pupils (50% boys) from attending the first year (15–17 years) (*n* = 4410) and second year (16–18 years) (*n* = 1828) of upper secondary school, without missing information on school grade and family economy (the main adjustment variables).

### Measures

#### Symptoms of anxiety and depression

Measures of anxiety and depression symptom load were based on ten items from the screening instrument Hopkins Symptom Checklist-25 [19]. The items were divided into measures of depression (six items which constitute the “Depressive Mood Inventory”) [20, 21], and anxiety (four items). The items from the Depressive Mood Inventory have been validated in clinical studies [20], while the current combination of anxiety items has not been validated. The pupils answered how often they had been bothered with each of the ten symptoms during the last week. Each item was answered on a four- point scale ranging from “not at all” [1] to “very much” [4] (Table 1).

**Table 1:** Statements regarding mental health symptoms used in the Ungdata-survey and included in this study

Question: During the previous week, have you ever had troubles with any of the following:	
Statements related to depression	
1. Feeling low in energy, slowed down	
2. Difficulties falling asleep, staying asleep	
3. Feeling blue	
4. Felt hopeless about the future	
5. Felt tense or keyed up	
6. Worrying too much about things	
Statements related to anxiety	
1. Suddenly scared for no reason	
2. Feeling fearful	
3. Faintless, dizziness or weakness	
4. Nervousness or shakiness inside	

Separate measures for depressive and anxiety symptoms were constructed by adding up the scores (1 to 4) on all the items covering each dimension (6 items for depression and 4 for anxiety) and dividing it by the number of completed items, given response to at least half of the statements for each scale. This resulted in two mean symptom scale scores, one for depression and one for anxiety, each ranging from 1 to 4. Cronbach’s alpha was 0.90 for the depression scale score, and 0.85 for the anxiety scale score. The mean scale scores were used to divide the boys and the girls into four groups reflecting the gender-specific symptom level of depression and anxiety (quartiles, labeled I - IV), where the first quartiles (I) represent the lowest symptom levels and the fourth quartiles (IV) represent the highest symptom levels.

#### Alcohol consumption

Alcohol consumption was measured using the following three questions: 1) “How old were you when you had your first full unit of alcohol, or drink?”; 2) “Do you ever drink any form of alcohol?”, response alternatives “never”, “have only tasted it a few times”, “now and then, but not as frequent as once a month”, “quite regular 1–3 times every month” and “Every week”; and 3) “If you consider the previous six months, how many times have you consumed enough alcohol to feel intoxicated?”, response alternatives “never”, “once”, “2–4 times”, “5–10 times” and “more than 10 times”.

We constructed the following dichotomous alcohol variables based on the questions above: *Early onset of alcohol consumption* (first unit of alcohol consumed before the age of 15), *frequent consumption* (answers “1–3 times every month” or “every week”) and *frequent intoxication* (answers “5–10 times” or “more than 10 times” during the previous six months).

#### Background variables

Background variables included sex, school year (first or second year of secondary school) and family socioeconomic status. The latter was measured by the question “Has your family’s economy been good or bad over the previous two years?” and answered on a five-point scale.

### Analysis

We used logistic regression analyses to examine the association between anxiety and depression symptom levels and each of the three dichotomized alcohol-related variables. In the main analyses, anxiety and depression symptom levels were assessed in gender-specific quartiles and included as categorical variables in separate analyses for each dimension. The analyses were stratified by sex and adjusted for school year and family socioeconomic status. Results from logistic regression analyses were used to estimate the predicted prevalences of early onset of alcohol drinking, frequent consumption and frequent intoxications according to symptom levels. For each quartile of symptoms of anxiety and depression, we also calculated differences in the predicted prevalences (average marginal effects) and corresponding odds ratios for each of the alcohol-related variables, with the first quartile as reference.

To examine if the relationships between anxiety and depression symptom load and alcohol consumption were different for boys and girls, for students in different school years or with different socioeconomic background, we tested for statistical interaction in the analyses (by including an interaction term between anxiety and depression symptoms and sex, anxiety and depression symptoms and school year and between anxiety and depression symptoms and family economy). Precision was measured using 95% confidence intervals. All analyses were conducted with STATA 13.

## Results

Characteristics of the study sample are shown in Table 2. A complete presentation of the alcohol-related variables is provided in the Appendix (Table 4).

**Table 2:** Overview of the study sample from the Ungdata-survey of 2013 which was used in this study (students in upper secondary school without missing data for sex, school year or their family’s socioeconomic status, *n* = 6238)

	Boys *n* = 3111		Girls *n* = 3127		Total *n* = 6238	
	*n*	%	*n*	%	*n*	%
Depression symptoms (range 1–4), mean *(SD)*	1.8 *(0.7)*		2.3 *(0.8)*		2.1 *(0.8)*	
I. quartile (boys 1.00–1.33, girls 1.00–1.67)	961	31	789	25	1750	28
II. quartile (boys 1.40–1.67, girls 1.75–2.33)	577	19	958	31	1535	25
III. quartile (boys 1.75–2.17, girls 2.40–2.83)	764	25	616	20	1380	22
IV. quartile (boys 2.20–4.00, girls 3.00–4.00)	704	23	692	22	1396	22
Missing	105	3	72	2	177	3
Anxiety symptoms (range 1–4), mean *(SD)*	1.3 *(0.5)*		1.7 *(0.7)*		1.5 *(0.7)*	
I. quartile (boys 1.00–1.00, girls 1.00–1.00)	1521	49	763	24	2284	37
II. quartile (boys 1.00–1.00, girls 1.25–1.50)			952	30	952	15
III. quartile (boys 1.25–1.50, girls 1.66–2.00)	878	28	571	18	1449	23
IV. quartile (boys 1.66–4.00, girls 2.25–4.00)	531	17	732	23	1263	20
Missing	181	6	109	3	290	5
Onset of alcohol consumption before the age of 15 years						
Yes	954	31	884	28	1838	29
No	1755	56	1946	62	3701	59
Missing	402	13	297	10	699	11
Consumes alcohol at least 1–3 times each month						
Yes	849	27	885	28	1734	28
No	2185	70	2199	70	4384	70
Missing	77	2	43	1	120	2
Been intoxicated at least five times over the past six months						
Yes	620	20	609	19	1229	20
No	2404	77	2470	79	4874	78
Missing	87	3	48	2	135	2
Year of upper secondary school						
1st year (11th year of schooling/age 15–17)	2231	72	2179	70	4410	71
2nd year (12th year of schooling/age 16–18)	880	28	948	30	1828	29
Has your family’s economy been good or bad over the previous two years?						
It has been good the entire time	1384	44	1174	38	2558	41
It has been good most of the time	984	32	1035	33	2019	32
It has neither been good nor bad	555	18	694	22	1249	20
It has been bad most of the time	142	5	181	6	323	5
It has been bad the entire time	46	1	43	1	89	1

**Fig. 1:**
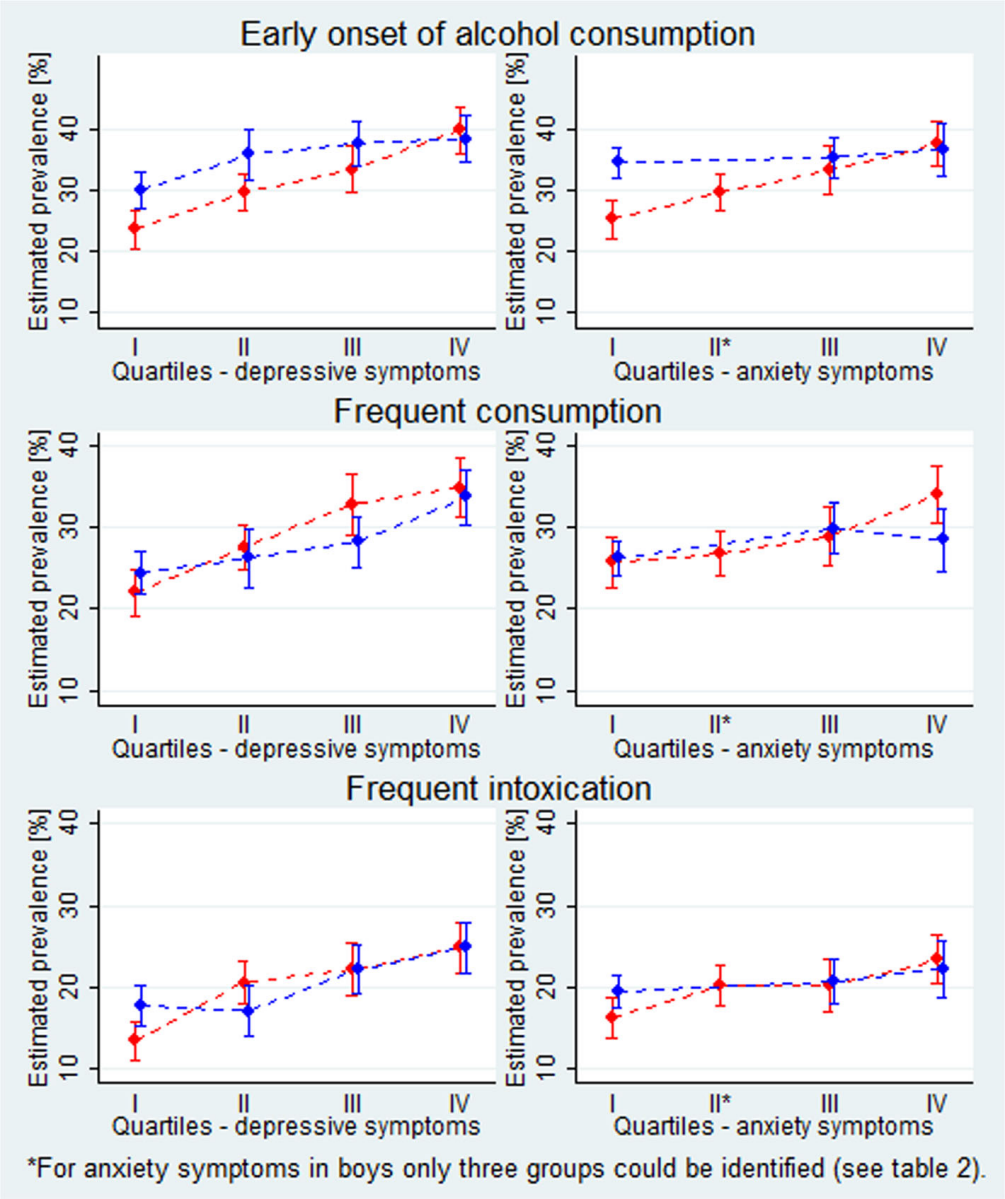
Estimated prevalences (in percentage points with 95% confidence intervals) of early onset of alcohol consumption, frequent consumption and frequent intoxication according to degree of symptoms of depression (*left*) and anxiety (*right*) in gender-specific quartiles (I = low symptom load, IV = high symptom load), for boys (*blue*) and girls (*red*), adjusted for school year and family socioeconomic status

Increased levels of depressive symptoms were associated with early onset of alcohol consumption, frequent consumption and frequent intoxication in both girls and boys. As symptom levels increased from low (first quartile - I) to high (fourth quartile – IV) a gradual increase was observed in the predicted prevalences of each of the alcohol variables (Fig. 1 – left column). Compared with girls in the first quartile, girls in the fourth quartile had substantial higher estimated prevalences of early onset of alcohol consumption (prevalence difference (PD) in percentage points 14, 95% CI 9–19), frequent consumption (PD 13, 95% CI 8–17) and frequent intoxication (PD 11, 95% CI 7–15). Similar, but weaker trends were observed for boys across all alcohol measures (Fig. 1 – left column), with estimated prevalence differences between 7 and 10. For increasing levels of anxiety symptoms, the associations with the alcohol variables were weaker than for depressive symptoms and only present in girls (Fig. 1 – right column). The largest difference between girls according to anxiety symptom load was observed for early onset of alcohol consumption, where girls in the fourth quartile had a 10 percentage points (95% CI 5–15) higher estimated prevalence of early onset of alcohol consumption than girls in the first quartile. Prevalence differences (average marginal effects) and corresponding odds ratios are shown in Table 3.

**Table 3:** Estimated prevalence differences (PD, in percentage points, representing average marginal effects) and corresponding odds ratios (OR) for alcohol drinking behaviours according to symptoms of anxiety and depression in gender-specific quartiles, with the first quartile serving as reference

	Boys		Girls	
	PD (95% CI)	OR (95% CI)	PD (95% CI)	OR (95% CI)
Depression symptoms				
Early onset				
2nd quartile (II)	6 (1–11)	1.3 (1.0–1.7)	5 (1–10)	1.3 (1.1–1.7)
3rd quartile (III)	8 (3–12)	1.4 (1.1–1.7)	9 (4–14)	1.5 (1.2–2.0)
4th quartile (IV)	8 (3–13)	1.4 (1.1–1.8)	14 (9–19)	1.9 (1.5–2.5)
Frequent consumption				
2nd quartile (II)	3 (−2–7)	1.1 (0.9–1.5)	6 (2–10)	1.4 (1.1–1.7)
3rd quartile (III)	4 (0–8)	1.2 (1.0–1.5)	11 (7615)	1.7 (1.4–2.2)
4th quartile (IV)	10 (5–14)	1.6 (1.3–2.0)	13 (8–17)	1.9 (1.5–2.4)
Frequent intoxication				
2nd quartile (II)	0 (−4–4)	1.0 (0.8–1.3)	7 (4–11)	1.7 (1.3–2.2)
3rd quartile (III)	5 (1–8)	1.3 (1.1–1.7)	8 (4–13)	1.8 (1.4–2.4)
4th quartile (IV)	7 (3–12)	1.6 (1.2–2.0)	11 (7–15)	2.1 (1.6–2.8)
Anxiety symptoms				
Early onset				
2nd quartile^a^ (II)	–	–	4 (−1–8)	1.2 (1.0–1.5)
3rd quartile (III)	1 (−3–5)	1.0 (0.9–1.3)	7 (2–12)	1.4 (1.1–1.8)
4th quartile (IV)	2 (−3–7)	1.1 (0.9–1.3)	10 (5–15)	1.6 (1.3–2.0)
Frequent consumption				
2nd quartile^a^ (II)	–	–	1 (−3–5)	1.0 (0.8–1.3)
3rd quartile (III)	4 (0–8)	1.2 (1.0–1.5)	3 (−2–8)	1.2 (0.9–1.5)
4th quartile (IV)	3 (−2–7)	1.2 (0.9–1.4)	8 (3–12)	1.5 (1.2–1.8)
Frequent intoxicaton				
2nd quartile^a^ (II)	–	–	3 (0–7)	1.3 (1.0–1.6)
3rd quartile (III)	2 (−2–5)	1.1 (0.9–1.4)	3 (−1–8)	1.3 (0.9–1.7)
4th quartile (IV)	3 (−1–7)	1.2 (0.9–1.6)	6 (2–10)	1.5 (1.1–1.9)

^a^For anxiety symptoms in boys only three groups could be identified (see Table 2)

Model adjusted for school year and family socioeconomic status

The differences in the alcohol-related behaviours between adolescents with high and low symptom load were generally larger for girls than for boys, but we found evidence for effect measure modification by sex only for the association between symptoms of anxiety and early onset of alcohol consumption and between symptoms of depression and frequent intoxication (*p*-values for statistical interaction with sex were 0.01 and 0.04).

We found no evidence for effect modification by school year, but we observed a general tendency towards stronger associations for first grade students compared with second grade students (*p*-values for statistical interaction between symptoms of depression/anxiety and school grade > 0.05).

## Discussion

### Summary of main findings

Increasing severity of depressive symptoms was associated with earlier onset of alcohol consumption, more frequent alcohol consumption and more frequent intoxication, also among students with mild or moderate symptoms. Increasing severity of anxiety symptoms was only substantially associated with the alcohol variables among girls. The associations between symptoms of anxiety and depression and early onset of alcohol consumption were stronger for girls than for boys.

### Strengths and weaknesses

The data used in this study was collected recently and provides up-to-date knowledge, which is particularly valuable in a world where the youth culture is constantly changing. The study included a large study sample that makes chance an unlikely explanation for the main findings. We used several measures of alcohol consumption, and several different questions to examine the association with mental distress properly. We do not know the reliability of the alcohol measures used in the present study, however previous research indicates that one can have confidence in adolescents’ survey self-reports on alcohol use and alcohol-related problems [22, 23]. Better data on mental health with exclusively validated instruments would have strengthened the findings. This is particularly the case with anxiety symptoms, where the weak associations with alcohol drinking patterns may be partly due to the current measure of symptoms. Because of missing or incomplete information about underlying factors such as socioeconomic status, health status or family-related problems, the analyses were conducted with limited adjustment. There were also varying levels of missing information on most of the study variables, and the restriction of our analyses to those with available data could have biased our results. However, analyses on imputed datasets were performed as sensitivity analyses (not shown), and these all showed similar effect estimates as in the presented results.

As we only used a subsample from the Ungdata survey (those with available information on anxiety symptoms), we ensured that the key figures in our sample were similar to those presented in reports for the entire Ungdata population [13] (including study variables, self-reported health and school satisfaction in subgroups according to sex and school grade).

The results might be representative of other adolescent groups beyond the adolescent population of Norway in 2013. Nevertheless, both adolescent mental illness and alcohol behavior is likely to be influenced by contextual factors and whether the results could be generalized to other European countries is questionable. There are particularly large differences in alcohol drinking patterns among adolescents from different countries and in different periods. The amount of alcohol consumed by Norwegian students is less than the European average, but as in the other Nordic countries Norwegian adolescents consume large amounts on few occasions and girls drink almost as much as boys [1, 14]. Recently, both the amount of alcohol consumed and drunkenness has decreased among 16-year olds in Norway as well as in the other Nordic countries [1]. Results may therefore also be relevant in a Nordic perspective.

This is a cross-sectional study, resulting in limitations regarding the questions of causality. It is not possible to disentangle whether anxiety or depression symptoms led to increased alcohol use or the other way around – both possible mechanisms according to literature [10, 24] – or whether the associations could be the result of other factors that are related to both mental health problems and alcohol behaviour [7, 8]. Examples of such factors could be genetic factors, personality, poor performance in school, social problems and other comorbid mental disorders, such as attention deficit hyperactivity disorder (ADHD) or conduct disorder [15].

### Interpretations

Our findings of an evident correlation between level of self-reported measures of depressive symptoms and alcohol consumption are in accordance with existing literature [15–17]. A recent Norwegian study of adolescents aged 17–19 found that frequent alcohol consumption and frequent intoxication were associated with increasing levels of depression symptoms [15]. An association between depressive symptoms and frequent alcohol consumption has also been found in American middle school students [10], suggesting that the relationship is present already in the beginning of adolescence. While other studies often report on associations between high levels of depressive symptoms (e.g. using cut-off values to define the adolescents that are likely to be depressed) and alcohol behavior/consumption, the present study indicate that the co-occurrence of anxiety or depression symptoms and increased alcohol use can be seen in a large proportion of the youth population, also for symptoms levels and alcohol drinking behaviours which usually are considered part of normal adolescence.

Our results indicated that anxiety symptoms were more weakly related to alcohol consumption. Again, this is in accordance with the recent Norwegian study in adolescents 17–19 years old [15]. In this study, anxiety symptoms were associated with heavy alcohol consumption and a measure of problematic alcohol and drug use, but not with frequent intoxications. Associations were weaker than for symptoms of depression, inattention and hyperactivity and substantially reduced when adjusted for other mental health variables [15]. However, other studies have indicated a relationship between symptoms of anxiety and hazardous alcohol use during adolescence [25, 26]. In a review, Blumenthal et al. [27] concludes that the relationship differs according to the type of anxiety symptoms. While panic disorder and social anxiety seem to have a clear relationship with hazardous alcohol use, the link is less evident for generalized anxiety disorder, and there seems to be no relationship with separation anxiety. It is also possible that the relationships with symptoms of anxiety increases with age [28], perhaps primarily due to increased availability of alcohol, and increase in the inclinations to use alcohol as a mean to cope with anxiety symptoms.

We found a tendency for stronger associations between anxiety and depression symptoms and hazardous alcohol use for girls compared with boys. This is in accordance with previous research for symptoms of anxiety [29, 30], while research on sex differences regarding associations with symptoms of depression alone is scarce. The Norwegian study on adolescents aged 17–19 years did not reveal any important sex differences [15], but this study did not include a measure of “early onset”, for which sex seemed to have some differential impact in our study. This discrepancy could also be explained by the adolescents in our study being somewhat younger. We know that the level of mental health symptoms increases earlier for those adolescent girls with early sexual maturation [31]. Mature girls could be more likely to seek companionship with older boys/adolescents, where alcohol consumption is more common, thereby accelerating their exposure to alcohol. Also, we cannot rule out the possibility that a stronger relationship between depression and alcohol use in girls partially could be explained by sex differences in the accuracy of self-reported depression, although no important sex differences were found in recent validation studies on similar depression scales among Norwegian and Danish adolescents [32].

## Conclusions

This study points to adolescents with depressive symptoms as a group that are more likely to have unhealthy patterns of alcohol drinking. Preventive strategies at all levels could possibly profit from a common approach to mental health and alcohol use, in particular for girls in mid-adolescence. More research is needed to further examine the causal relationship between alcohol consumption and mental health problems, and to identify strategies and targets for prevention.

## Appendix

**Table 4:** Detailed presentation of the alcohol-related variables used in the Ungdata-survey

	Boys *n* = 3111		Girls *n* = 3127		Total *n* = 6238	
	*n*	%	*n*	%	*n*	%
How old were you when you had your first full unit of alcohol, or drink?						
I have never consumed that much alcohol	736	24	701	22	1437	23
Less than 10 years old	96	3	16	0.5	112	2
10 years old	31	1	11	0.4	42	0.7
11 years old	29	1	15	0.5	44	0.7
12 years old	139	4	73	2	212	3
13 years old	249	8	278	9	527	8
14 years old	410	13	491	16	901	14
15 years old	569	18	723	23	1292	21
16 years old	367	12	437	14	804	13
17 years old	56	2	61	2	117	2
18 years old or older	27	1	24	0.8	51	0.8
Missing	402	13	297	10	699	11
Do you ever drink any form of alcohol?						
Never	701	23	595	19	1296	21
Have only tasted it a few times	575	18	541	17	1116	18
Now and then, but not as frequent as once a month	909	29	1063	34	1972	32
Quite regular 1–3 times every month	673	22	762	24	1435	23
Every week	176	6	123	4	299	5
Missing	77	2	43	1	120	2
If you consider the previous six months, how many times have you consumed enough alcohol to feel intoxicated?						
Never	1376	44	1219	39	2595	42
Once	412	13	442	14	854	14
2–4 times	616	20	809	26	1425	23
5–10 times	356	11	355	11	711	11
10 times or more	264	8	254	8	518	8
Missing	87	3	48	2	135	2
